# Supplementary material for: Phytochemical Characterization of Cannabis sativa L. Chemotype V Reveals Three New Dihydrophenanthrenoids That Favorably Reprogram Lipid Mediator Biosynthesis in Macrophages
Source: Plants (Basel). 2022 Aug 16;11(16):2130. doi: 10.3390/plants11162130 (PMC9414986; doi:10.3390/plants11162130)
Supplement: Supplementary file 1 [file plants-11-02130-s001.zip › plants-1846845-supplementary.pdf]

Phytochemical characterization of *Cannabis sativa* L. chemotype V reveals three new dihydrophenanthrenoids that favourably reprogram lipid mediator biosynthesis in macrophages

Stefano Salamone[a,b,#], Lorenz Waltl[c,#] Anna Pompignan[a], Gianpaolo Grassi[d], Giuseppina Chianese[e], Andreas Koeberle[c*], Federica Pollastro [a,b *]

[a] Department of Pharmaceutical Sciences, University of Piemonte Orientale, 28100 Novara, Italy

[b] PlantaChem Srls, 28100 Novara, Italy

[c] Michael Popp Institute and Center for Molecular Biosciences Innsbruck (CMBI), University of Innsbruck, 6020 Innsbruck, Austria

[d] Canvasalus Srl, 35043 Monselice, Italy

[e] Department of Pharmacy, University of Naples Federico II, 80131 Naples, Italy

[#] Authors contributed equally

* Correspondence: Andreas.Koeberle@uibk.ac.at, Tel.: +43 512 507-57903 federica.pollastro@uniupo.it, Tel.: +39 321 375 744

SUPPORTING INFORMATION:

Figure S1. ^{1}H NMR spectrum (400 MHz) of compound 5 in C_3D_6O.

Figure S2. ^{13}C NMR spectrum (100 MHz) of compound 5 in C_3D_6O.

Figure S3. HSQC 2D NMR spectrum (400 MHz) of compound 5 in C_3D_6O.

Figure S4. COSY 2D NMR spectrum (400 MHz) of compound 5 in C_3D_6O.

Figure S5. NOESY 2D NMR spectrum (400 MHz) of compound 5 in C_3D_6O.

Figure S6. HMBC 2D NMR spectrum (600 MHz) of compound 5 in C_3D_6O.

Figure S7. ^{1}H NMR spectrum (400 MHz) of compound 9 in C_3D_6O.

Figure S8. ^{13}C NMR spectrum (100MHz) of compound 9 in C_3D_6O.

Figure S9. HSQC 2D NMR spectrum (400 MHz) of compound 9 in C_3D_6O.

Figure S10. COSY 2D NMR spectrum (400 MHz) of compound 9 in C_3D_6O.

Figure S11. NOESY 2D NMR spectrum (600 MHz) of compound 9 in C_3D_6O.

Figure S12. HMBC spectrum (400 MHz) of compound 9 in C_3D_6O.

Figure S13. ^{1}H NMR spectrum (400 MHz) of compound 10 in C_3D_6O.

Figure S14. ^{13}C NMR spectrum (100MHz) of compound 10 in C_3D_6O.

Figure S15. HSQC 2D NMR spectrum (400 MHz) of compound 10 in C_3D_6O.

Figure S16. COSY 2D NMR spectrum (400 MHz) of compound 10 in C_3D_6O.

Figure S17. HMBC 2D NMR spectrum (600 MHz) of compound 10 in C_3D_6O.

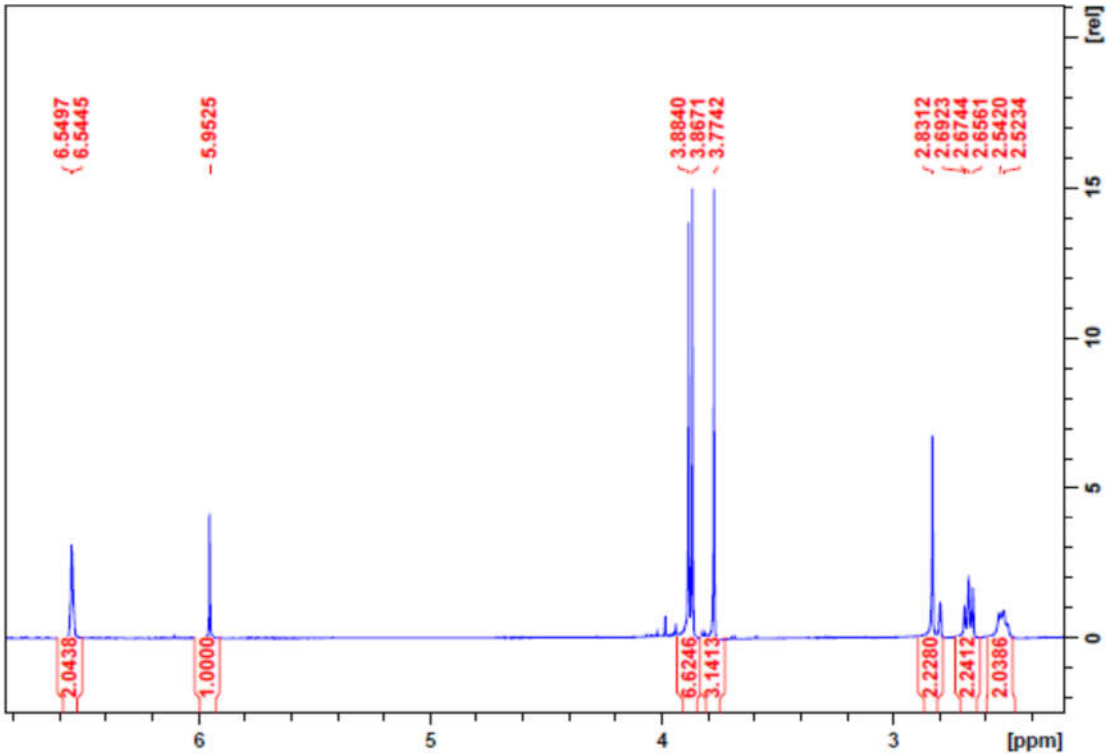

Figure S1. ^{1}H NMR spectrum (400 MHz) of compound 5 in C_3D_6O.

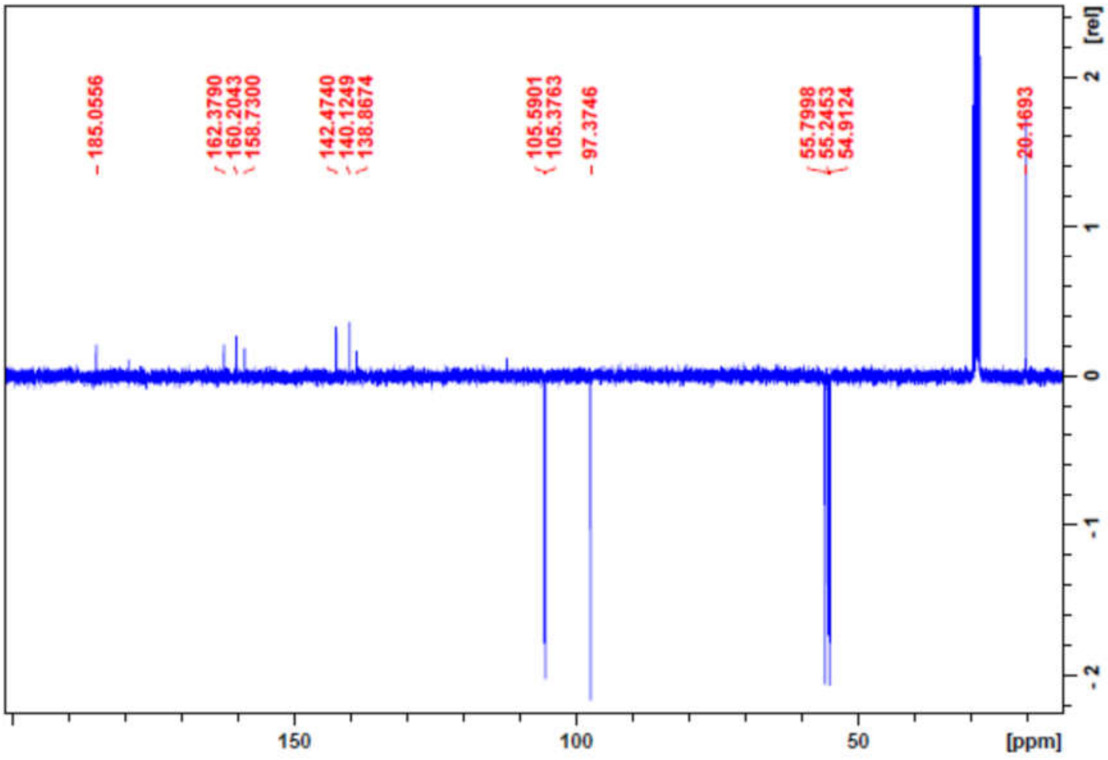

Figure S2. ^{13}C NMR spectrum (100 MHz) of compound 5 in C_3D_6O.

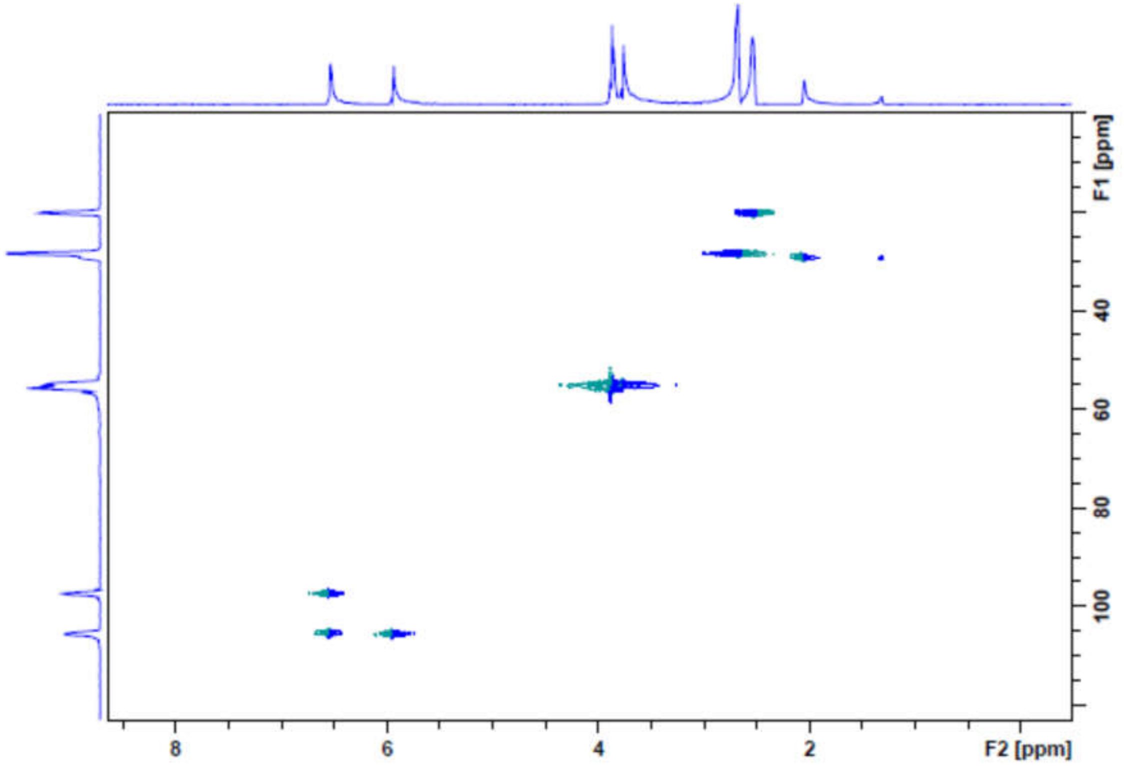

Figure S3. HSQC spectrum (400 MHz) of compound 5 in C_3D_6O.

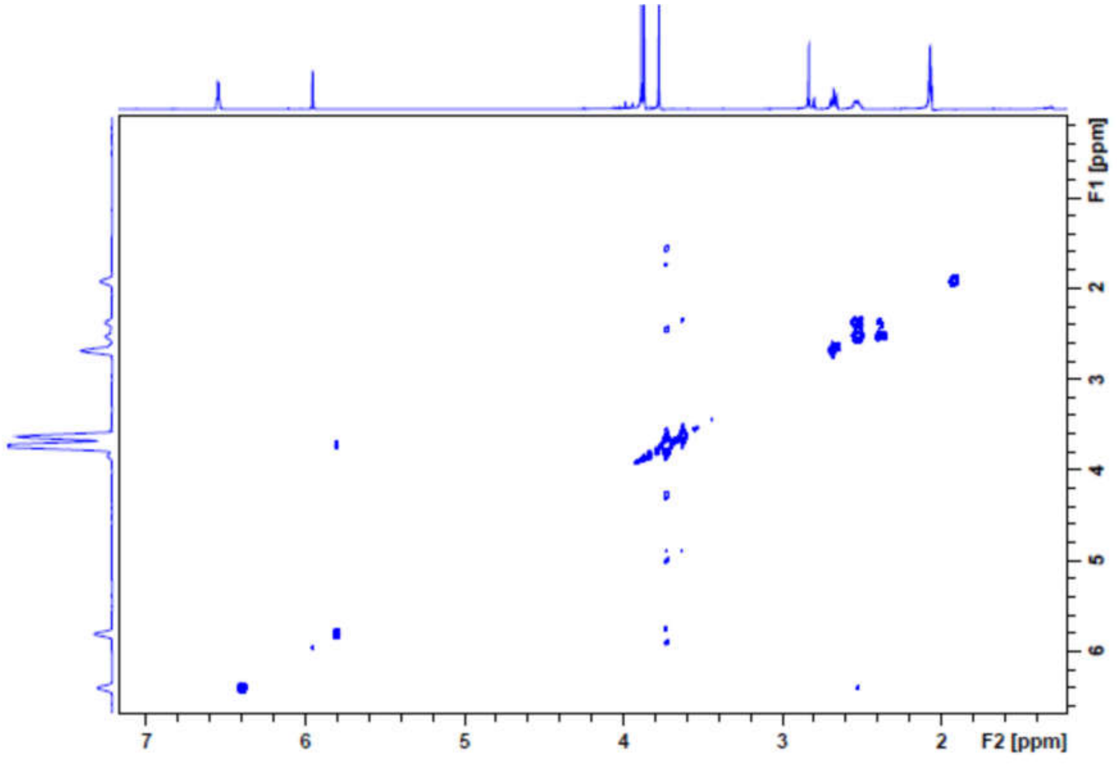

Figure S4. COSY spectrum (400 MHz) of compound 5 in C_3D_6O.

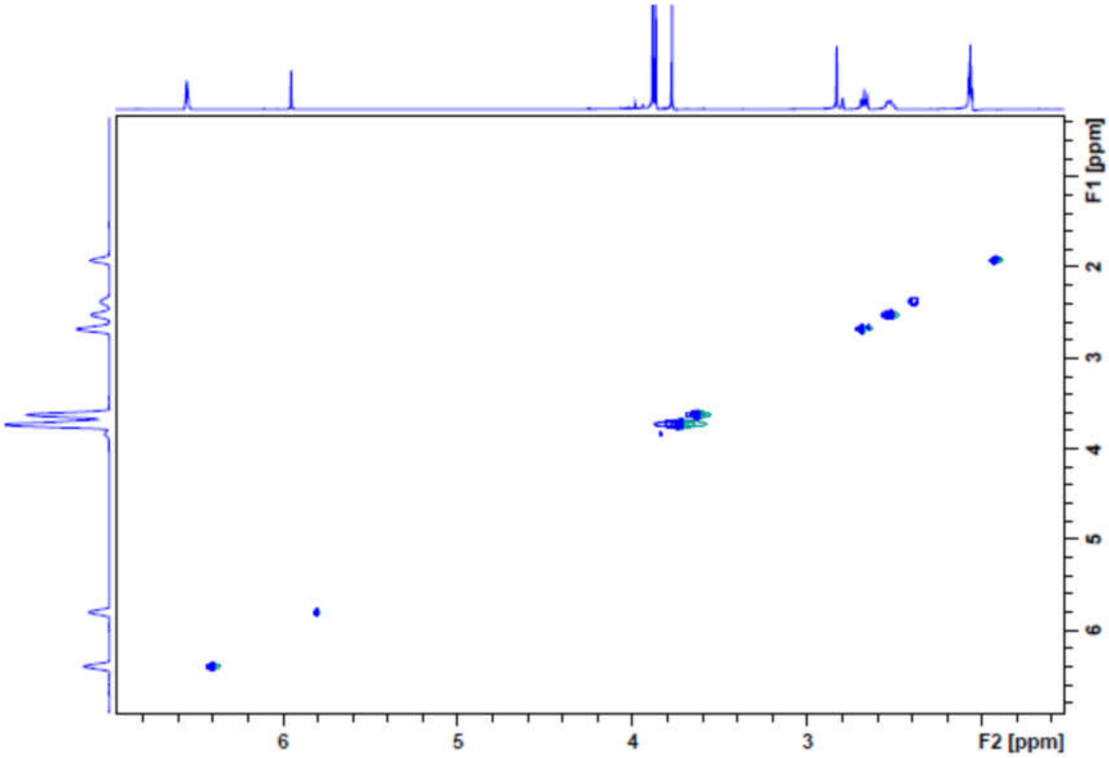

Figure S5. NOESY spectrum (400 MHz) of compound 5 in C_3D_6O.

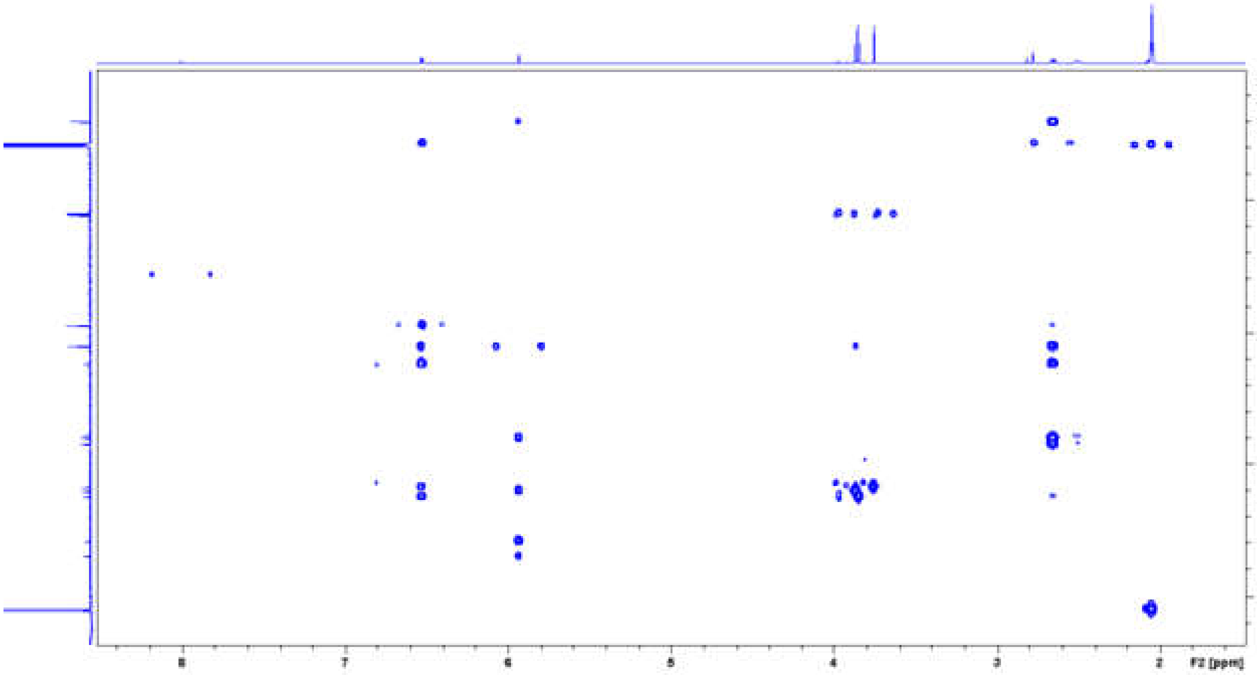

Figure S6. HMBC 2D NMR spectrum (600 MHz) of compound 5 in C_3D_6O.

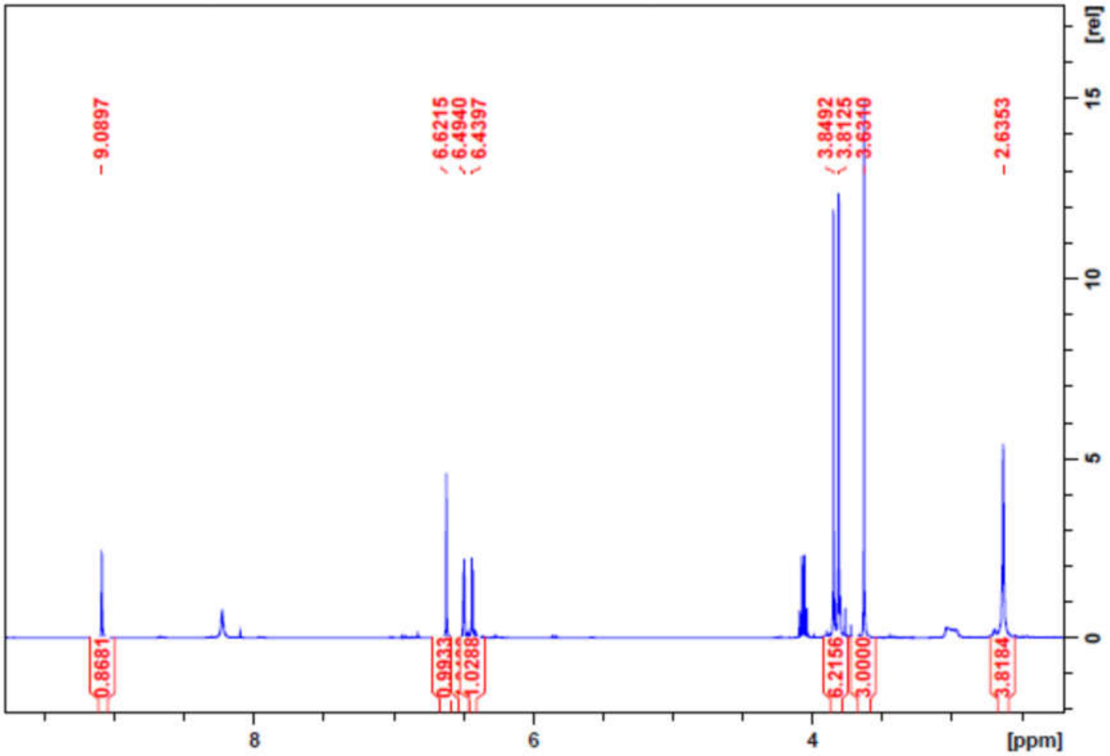

Figure S7. ^{1}H NMR spectrum (400 MHz) of compound 9 in C_3D_6O.

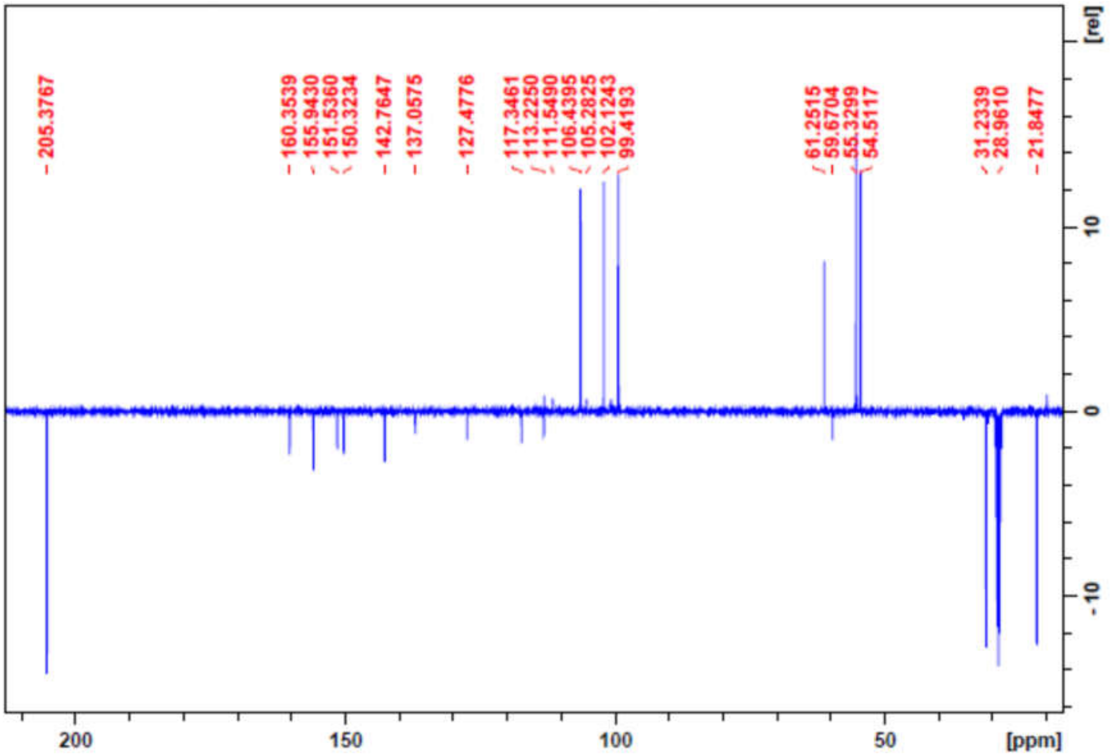

Figure S8. ^{13}C NMR spectrum (100MHz) of compound 9 in C_3D_6O.

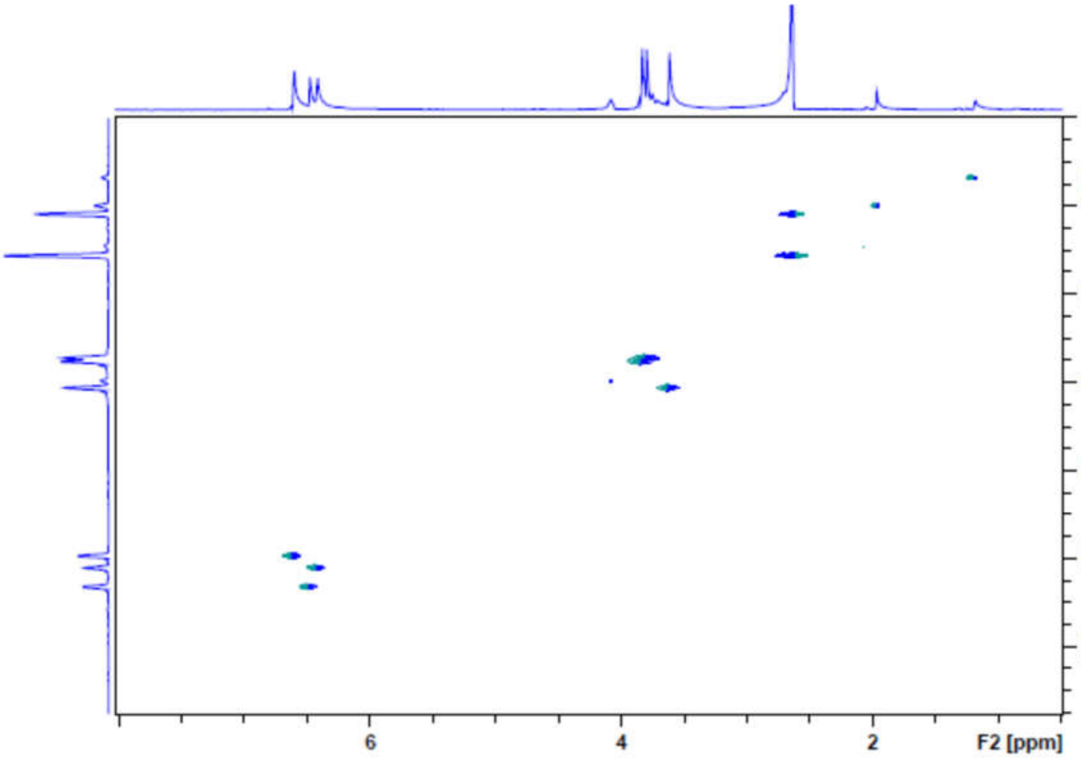

Figure S9. HSQC 2D NMR spectrum (400 MHz) of compound 9 in C_3D_6O.

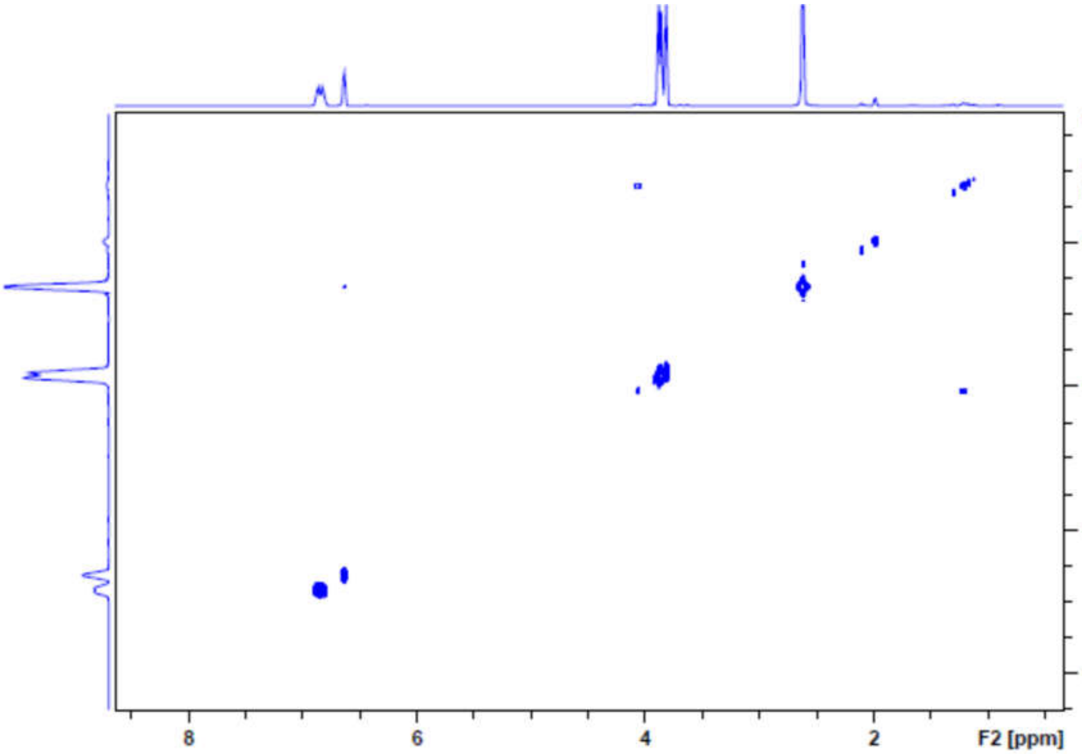

Figure S10. COSY 2D NMR spectrum (400 MHz) of compound 9 in C_3D_6O.

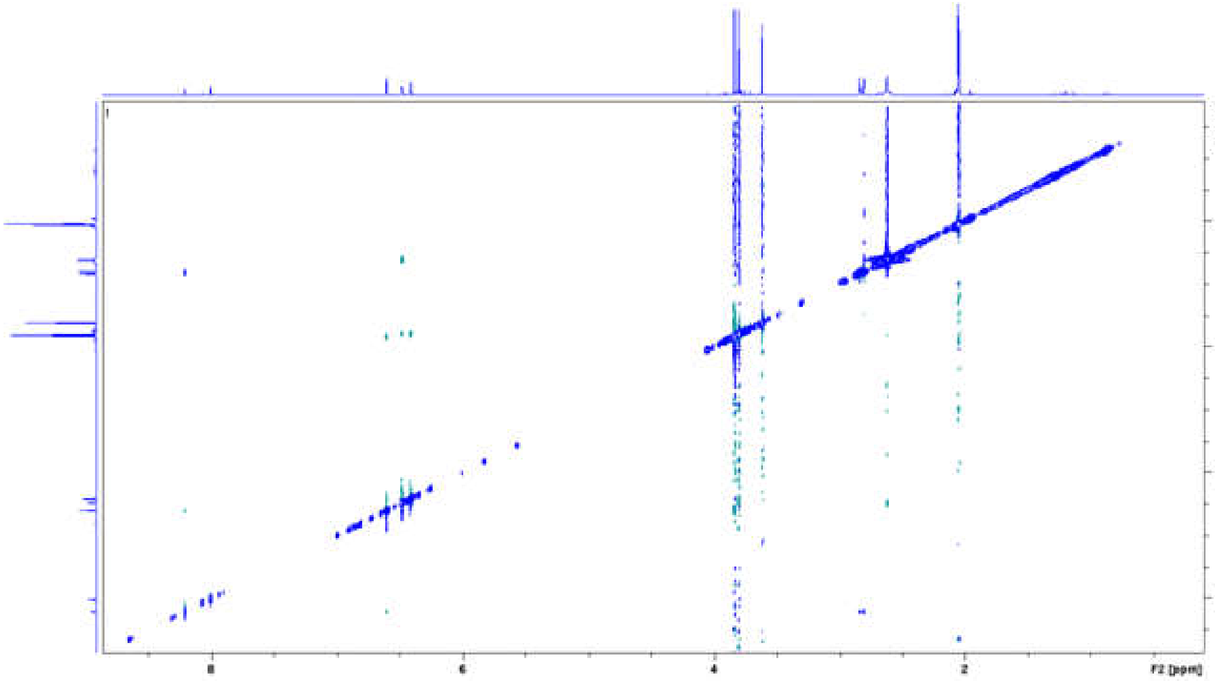

Figure S11. NOESY 2D NMR spectrum (600 MHz) of compound 9 in C_3D_6O.

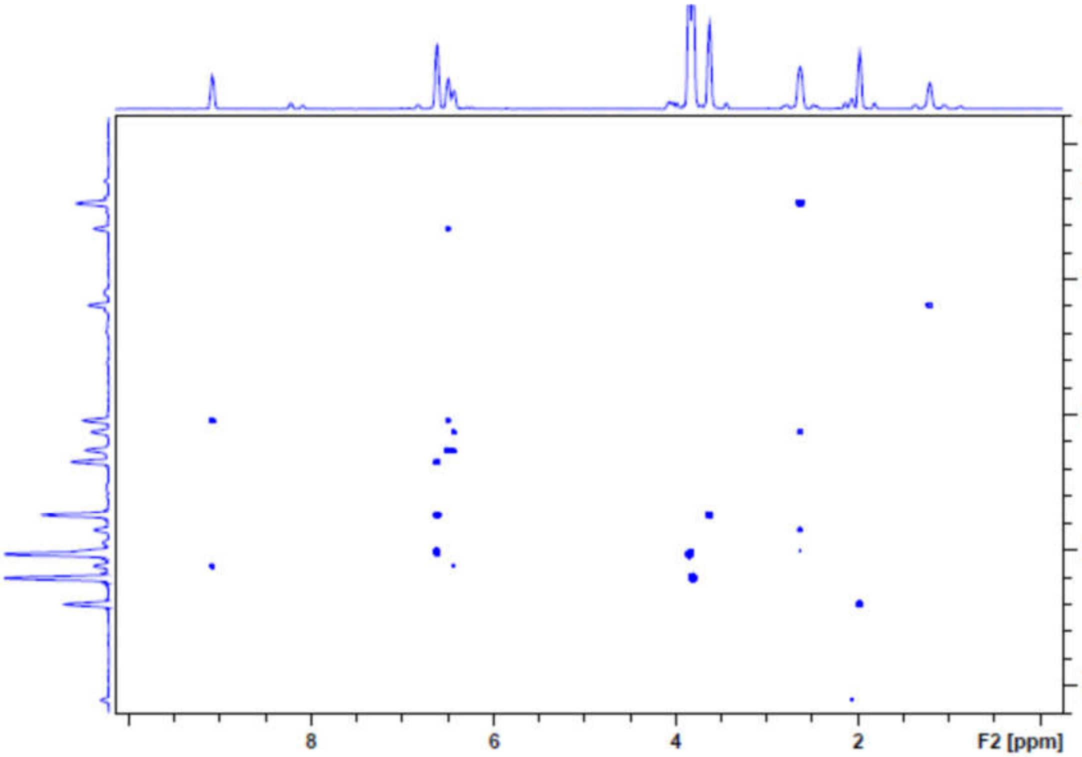

Figure S12. HMBC 2D NMR spectrum (400 MHz) of compound 9 in C_3D_6O.

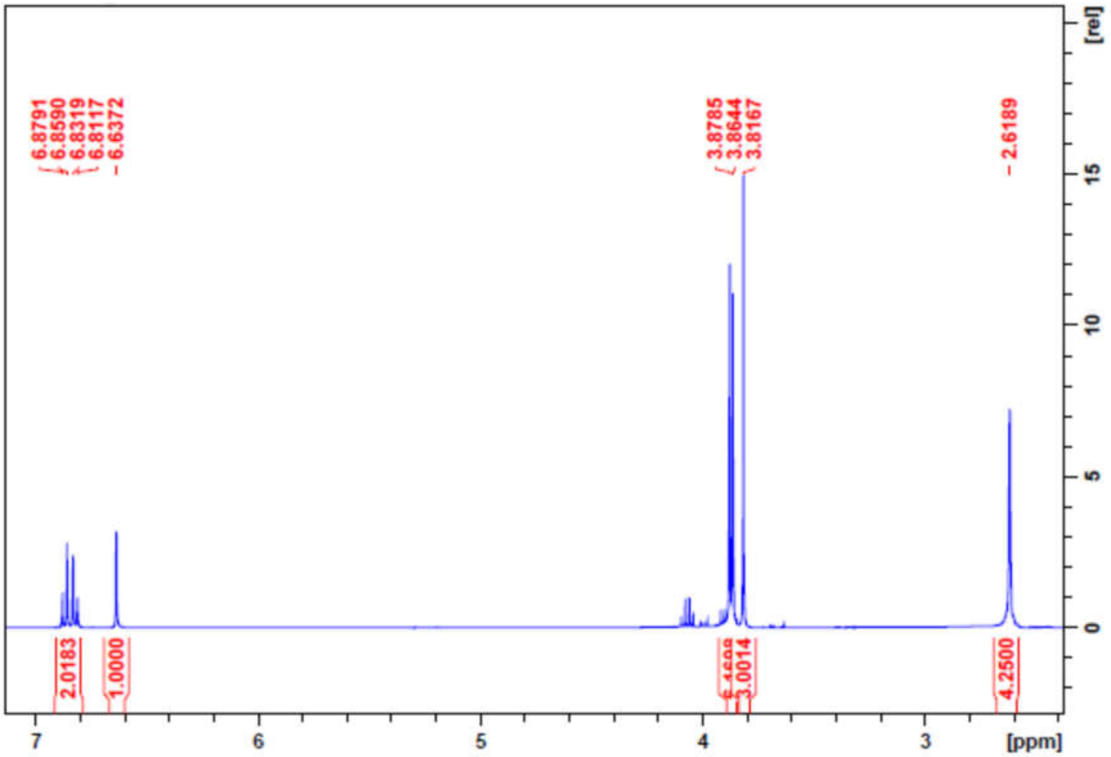

Figure S13. ^{1}H NMR spectrum (400 MHz) of compound 10 in C_3D_6O.

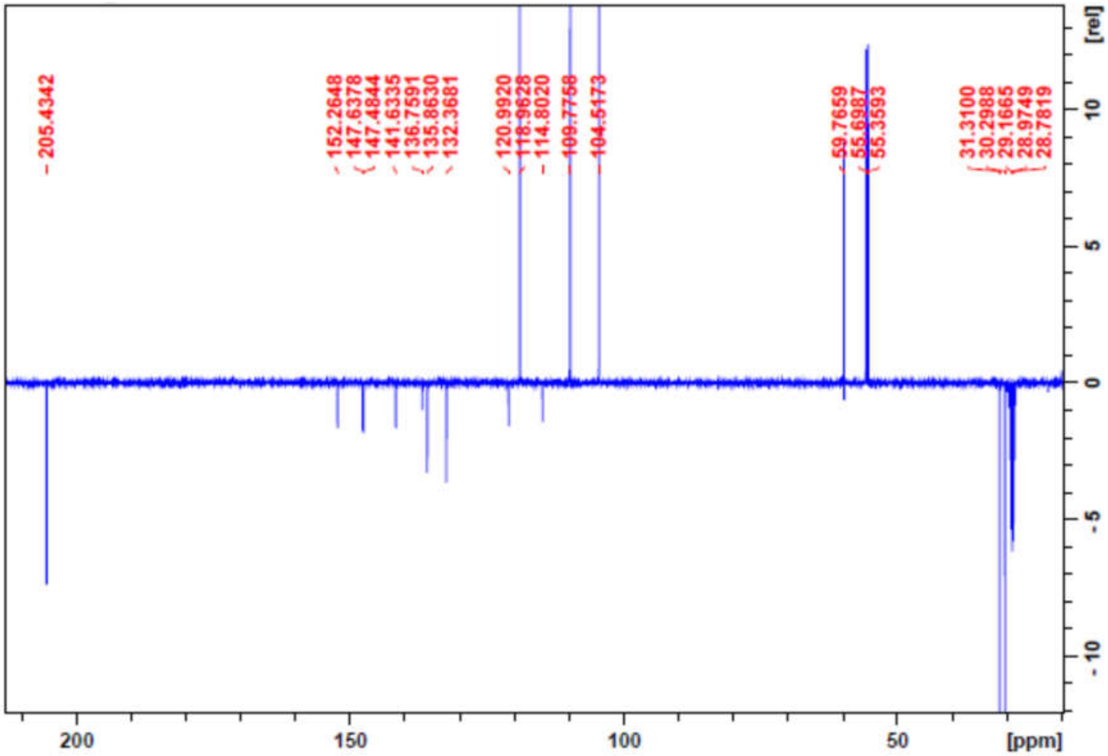

Figure S14. ^{13}C NMR spectrum (100MHz) of compound 10 in C_3D_6O.

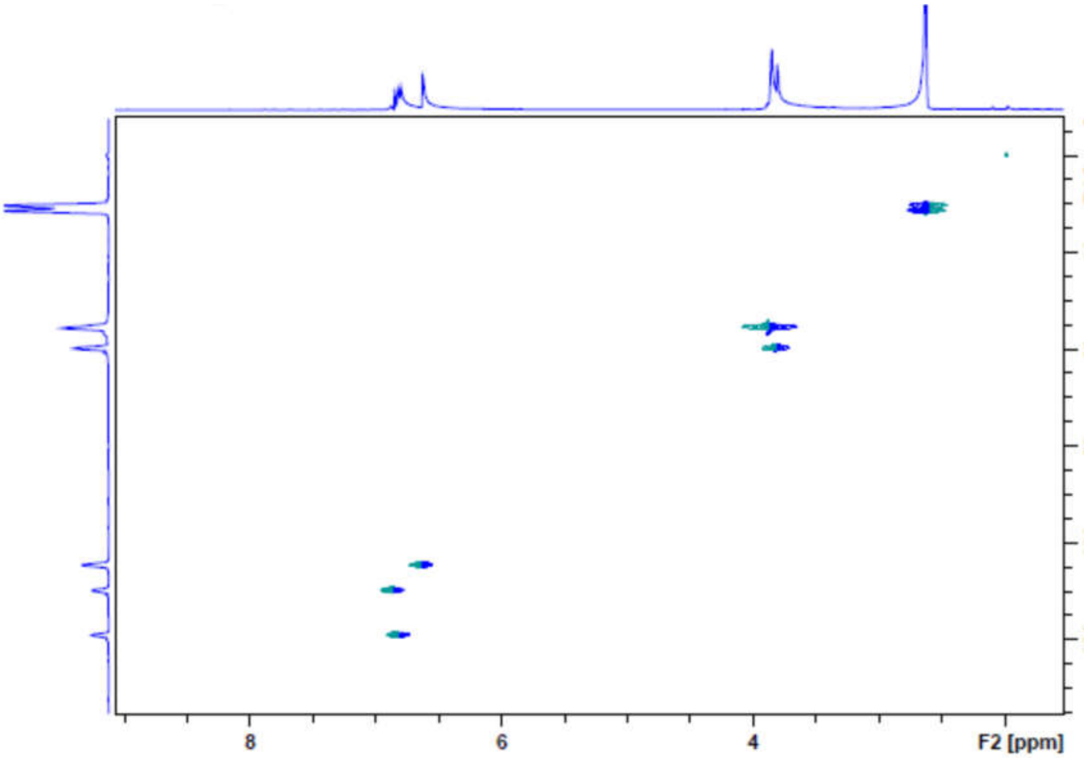

Figure S15. HSQC 2D NMR spectrum (400 MHz) of compound 10 in C_3D_6O.

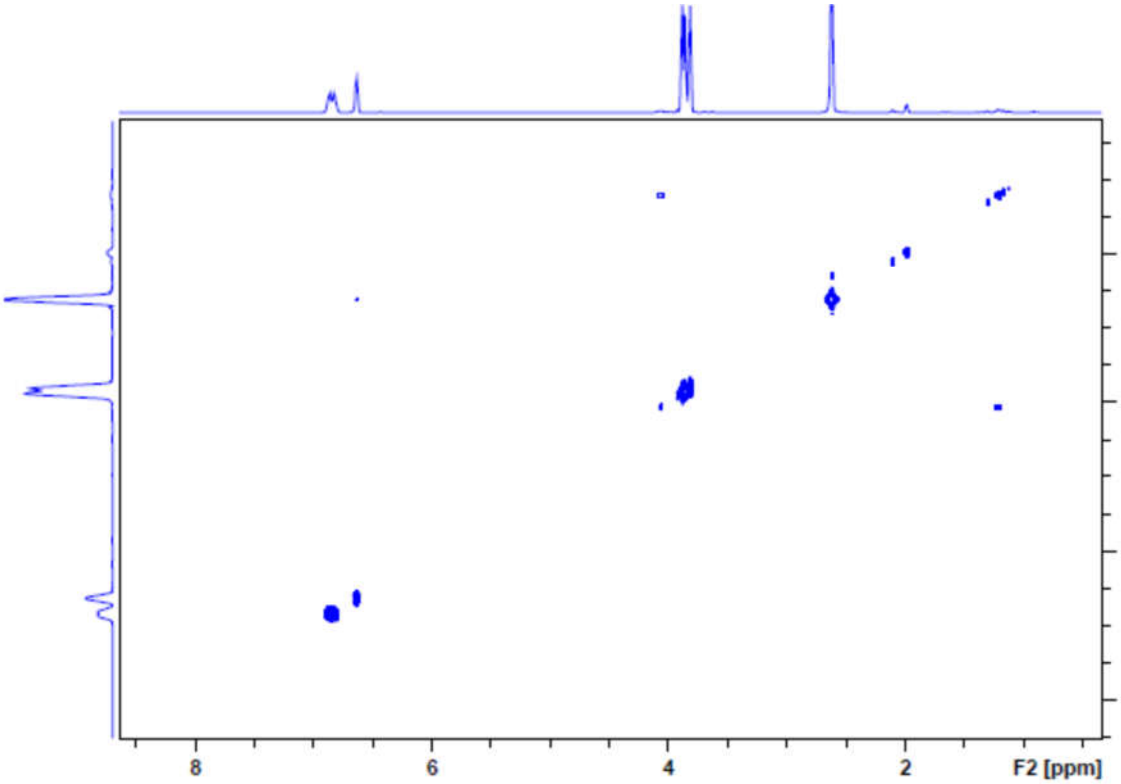

Figure S16. COSY 2D NMR spectrum (400 MHz) of compound 10 in C_3D_6O.

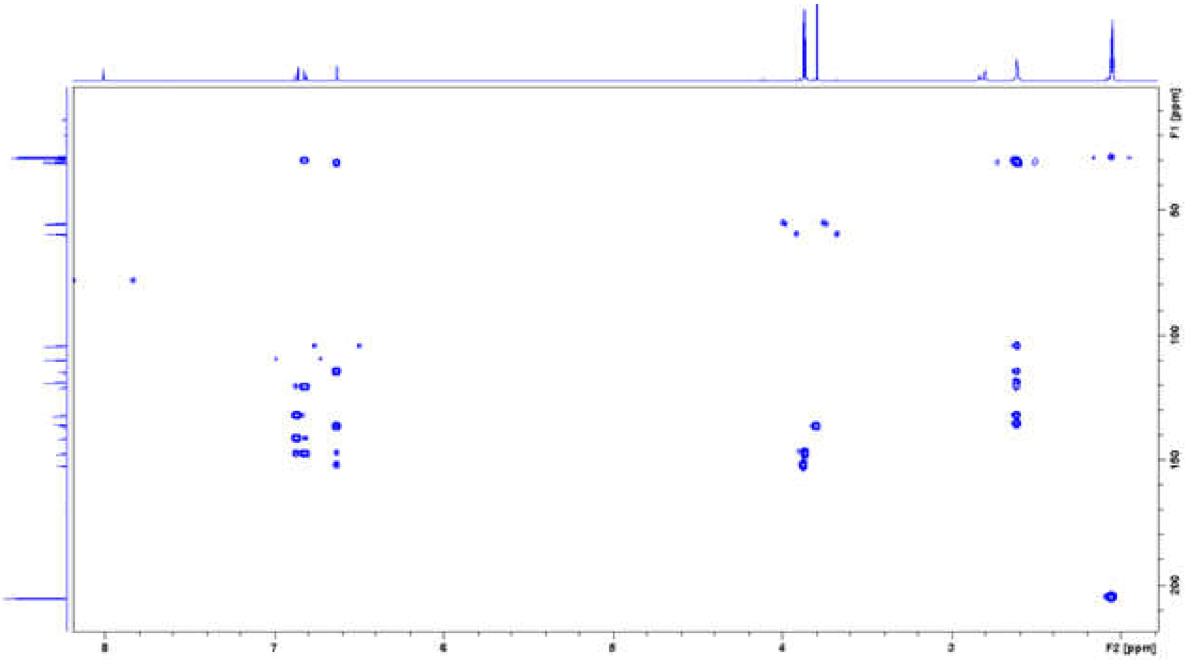

Figure S17. HMBC 2D NMR spectrum (600 MHz) of compound 10 in C_3D_6O.
